# The T stage of esophageal cancer can be effectively predicted by muscularis propria thickness and muscularis propria + mucosa thickness under ultrasonic gastroscopy

**DOI:** 10.1111/1759-7714.14722

**Published:** 2022-11-15

**Authors:** Yongkui Yu, Xiufeng Wei, Xiankai Chen, Haomiao Li, Qi Liu, Haibo Sun, Wei Wang, Lifeng Wang, Yin Li, Wenqun Xing

**Affiliations:** ^1^ Department of Thoracic Surgery The Affiliated Cancer Hospital of Zhengzhou University/Henan Cancer Hospital Zhengzhou China; ^2^ Department of Thoracic Surgery Beijing Chui Yang Liu Hospital Beijing China; ^3^ Department of Thoracic Surgery Cancer Hospital of the Chinese Academy of Medical Sciences Beijing China; ^4^ Radiology Department The Affiliated Cancer Hospital of Zhengzhou University/Henan Cancer Hospital Zhengzhou China

**Keywords:** esophageal cancer, T stage, thickness of the mucosa, thickness of the muscularis propria, treatment strategy

## Abstract

**Objectives:**

The latest version of the National Comprehensive Cancer Network recommends neoadjuvant therapy followed by surgical treatment or radical chemoradiotherapy for patients with cT3N0M0. Neoadjuvant therapy can improve the prognosis of patients with locally advanced esophageal cancer. Therefore, the evaluation or prediction of T stage is particularly important because the treatment could differently affect the prognosis. Here, we establish a model to predict the T stage of patients with T2‐3N0M0 to help choose the best treatment strategy.

**Methods:**

From 1637 patents with esophageal cancer, we enrolled 48 patients and performed least absolute shrinkage and selection operator regression to screen for independent factors influencing pathological T stage. We, then, trained the decision tree to obtain the decision tree diagram and divided the T stages obtained by different methods into two categories, T2 and T3, for survival analysis.

**Results:**

A total of 21 and 27 cases were predicted to be T2 and T3, respectively, under ultrasonic gastroscopy, 19 and 29 under magnetic resonance imaging, and 22 and 26 under pathological examination. Multivariate logistic regression analysis revealed that the muscularis propria thickness (MPT) (*p* = 0.0097) and the muscularis propria + mucosa thickness (MPMT) in the largest tumor cross‐section (*p* = 0.0239) were independent influencing factors. We plotted a decision tree diagram with these two factors. MPT in the largest tumor cross‐section >1.3 mm could be judged as pT3; if ≤1.3 mm, MPMT should be considered a thickness ≥1.7 mm could be judged as pT2 (otherwise pT3). Corresponding survival analysis was performed according to the T stage under different examination modalities.

**Conclusion:**

MPT in the largest tumor cross‐section and MPMT in the largest tumor cross‐section are independent predicting factors of pathological T stage.

## Graphical abstract

Of the 1637 patients with esophageal cancer, only 48 patients met our eligibility criteria, for patients with preoperative cT2 or cT3 tumors, the likelihood of postoperative T3 was 54%. When the thickness of the muscularis propria in the largest cross‐section of the tumor was >1.3 mm, it could be judged as pT3, but when the thickness of the muscularis propria in the largest cross‐section of the tumor was ≤1.3 mm, the thickness of the muscularis propria + mucosa in the largest cross‐section of the tumor should be considered. When the latter was ≥1.7 mm, it could be judged as pT2, otherwise pT3.

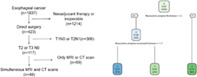

## INTRODUCTION

There are a variety of treatment regimens for esophageal cancer. The treatment regimen is selected according to empirically set staging guidelines, and the treatment modes will vary for different stages. This is especially true for patients with T3N0 cancer, where the treatment strategy is completely different from others. According to the latest version of the National Comprehensive Cancer Network (NCCN) guidelines, for patients with clinical cT2N0M0 cancer, if the tumor is <3 cm in diameter and well differentiated, direct surgical treatment is recommended. If the tumor is >3 cm and poorly differentiated, neoadjuvant chemoradiotherapy followed by surgical treatment or radical chemoradiotherapy is advised. For patients with cT3N0M0, direct surgical treatment is not recommended, but the same treatment principle for patients with >3‐cm poorly differentiated cT2N0M0 cancer should be followed: neoadjuvant therapy followed by surgical treatment or radical chemoradiotherapy. For patients with locally advanced esophageal cancer, neoadjuvant therapy can improve their prognosis.^1^, ^2^, ^3^, ^4^, ^5^, ^6^, ^7^ Therefore, the evaluation or prediction of T stage is particularly important, because the treatment principles of cT2N0M0 and cT3N0M0 differ greatly and will also differently affect the prognosis of patients.

The muscularis propria and mucosa are important parts of the esophageal wall, with blood vessels, lymphatic vessels, and nerves running inside them. Some studies have found that for patients with esophageal cancer, the number of lymphatic plexi in and adjacent to the tumor is significantly higher than that in patients with no tumor.^8^, ^9^, ^10^ The number of lymphatic plexi in the mucosa adjacent to the tumor is an independent predictor of overall survival in patients with esophageal cancer (hazard ratio, 2.06; *p* = 0.0049).^11^ What will happen to the structures around the tumor after it grows? Specifically, what will happen to the lymphatic vessels, because lymphatic metastasis is the main metastasis route of esophageal squamous‐cell carcinoma? We assume that with a change in the lymphatic plexus, the thickness of the muscularis propria and that of muscularis propria + mucosa may change to a certain degree?

Motived by the above questions, we established a model to predict the T stage of patients with T2‐3N0M0 esophageal cancer to help choose the best treatment strategy for these patients.

## MATERIALS AND METHODS

We screened the patients with esophageal cancer admitted to the Department of Thoracic Surgery of Henan Cancer Hospital from January 4, 2016 to April 9, 2018. The study protocol was approved by the Ethics Committee of the Affiliated Cancer Hospital of Zhengzhou University (2014ys38). All patients underwent enhanced computed tomography (CT) of the chest and upper abdomen for preoperative evaluation (GE 64‐row), and/or enhanced magnetic resonance imaging (MRI) (Siemens 3.0 T), ultrasonic gastroscopy (Olympus, UM3R), cardiac ultrasound, abdominal color ultrasound, upper gastrointestinal angiography, pulmonary function, and laboratory tests. We selected patients who underwent direct surgical treatment while undergoing CT and MRI at stage T2 or T3 (i.e., the thoracolaparoscopic McKeown procedure). We included factors that may affect T stage, such as the thickness of the muscularis propria, the thickness of the mucosa, submucosa, and the thickness of the muscularis propria + mucosa in the largest cross‐section of the tumor measured under ultrasonic gastroscopy, T stage under MRI, and T stage by ultrasonic gastroscopy. We used the glmnet package in R to perform least absolute shrinkage and selection operator (LASSO) regression to screen for independent influencing factors of pathological T stage. The factors selected by LASSO regression were included in multivariate logistic regression analysis using the rms package to build a prediction model. We, then, trained the decision tree with the rpart package to obtain the decision tree diagram. The ggsurvplot package was used to divide the T stages obtained by different methods into two categories, T2 and T3, for survival analysis.

## RESULTS

**FIGURE 1:**
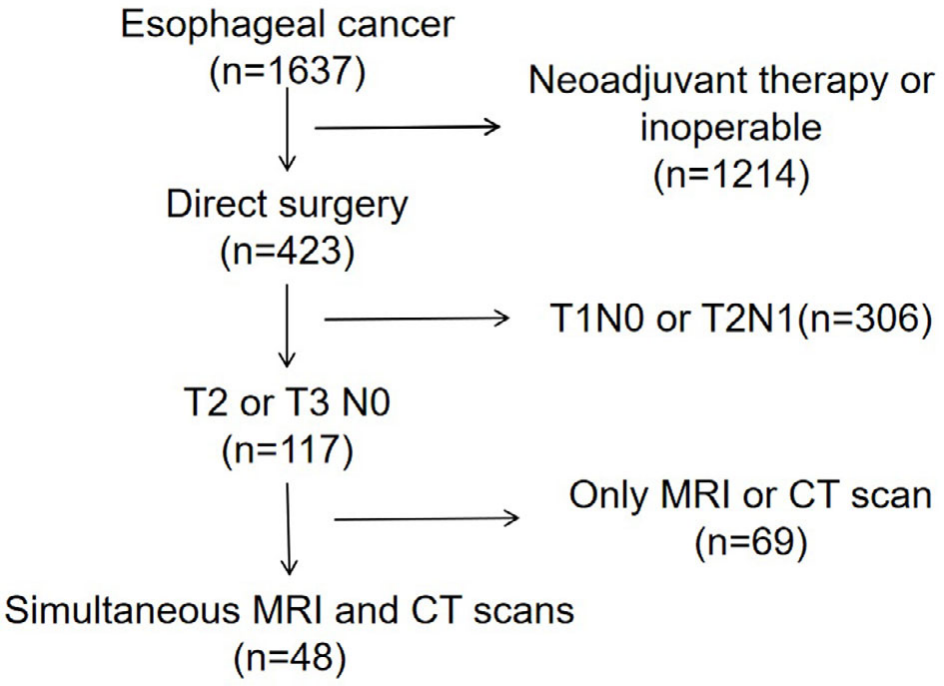
The flow chart of our study.

**FIGURE 2:**
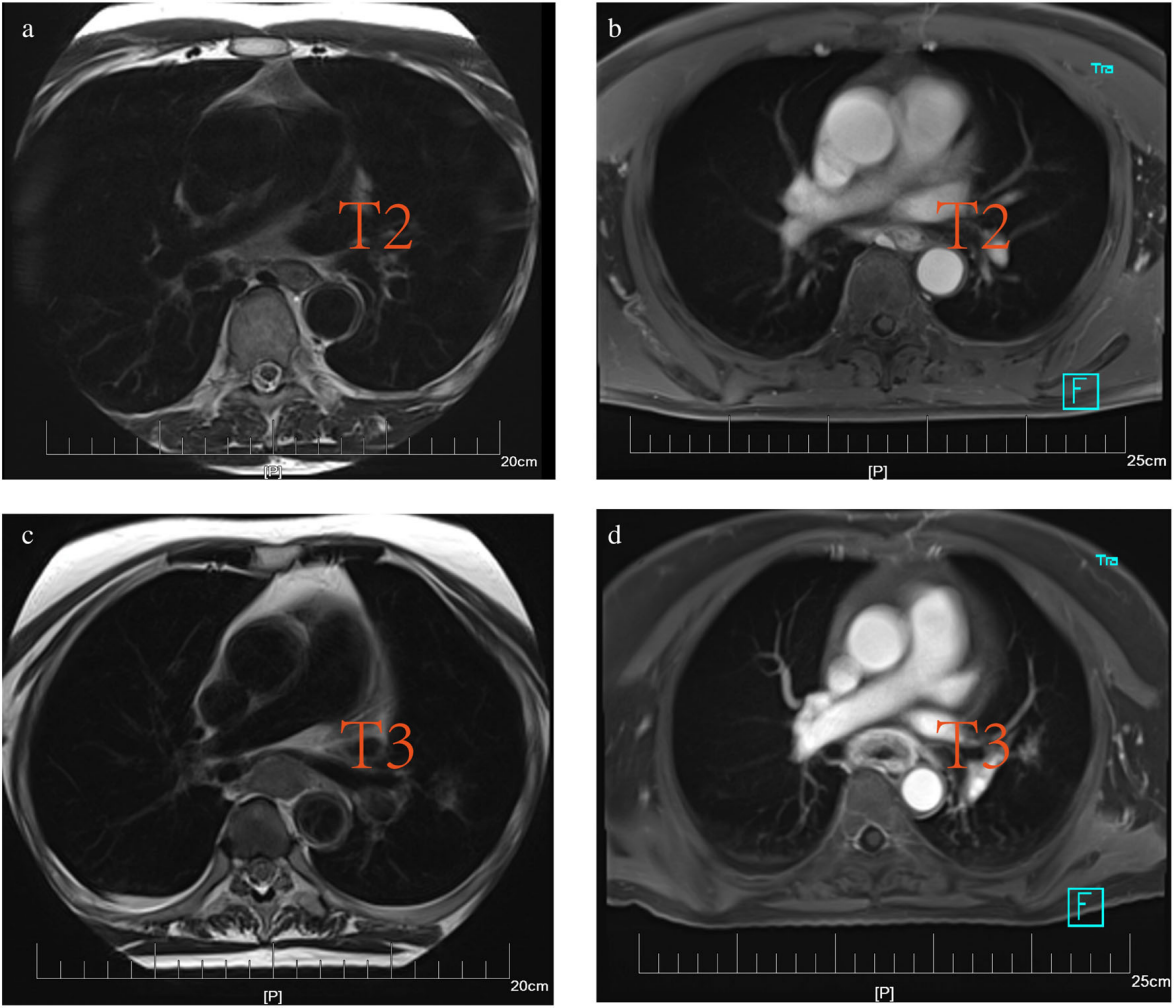
Magnetic resonance imaging (MRI) examination of patients with esophageal cancer ((a), (b) is the same patient, (c), (d) is the same patient). (a) T2 stage was diagnosed by T2WI sequence; (b) T2 was diagnosed under T1WI + c sequence; (c) T3 stage was diagnosed under T2WI sequence; (b) T3 stage was diagnosed under T1WI + c sequence.

**FIGURE 3:**
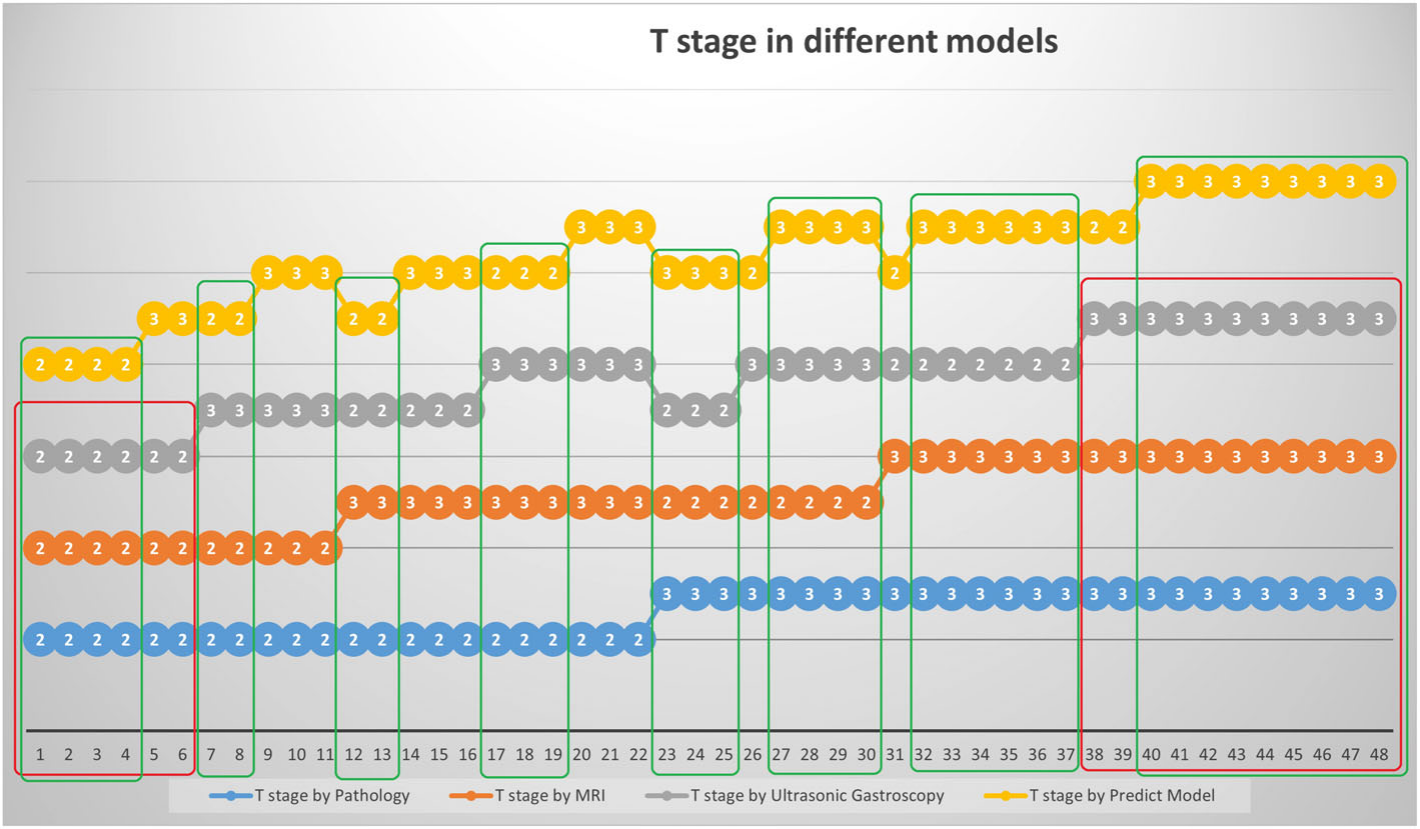
One‐to‐one correspondence of the T stage prediction methods for each patient under different staging modes. The patients in the green circle are those who were accurately judged by the prediction model, and the patients in the red circle are those whose ultrasound T stage and magnetic resonance imaging (MRI) T stage are the same and accurately judged.

Of the 1637 patients with esophageal cancer, only 48 patients met our eligibility criteria (Figure 1). Their ages ranged from 42 to 75 years old (63.04 ± 9.15) including 34 males and 14 females. Of these patients, 21 and 27 cancers were stage T2 and T3, respectively, under ultrasonic gastroscopy, 19 and 29 were T2 and T3 under MRI (Figure 2), and 22 and 26 were T2 and T3 under pathological examination. The patients' basic information is shown in Table 1. The one‐to‐one correspondence of MRI T stage, ultrasound T stage, pathology T stage, and predicted T stage is shown in Figure 3 and Table 2.

**TABLE 1:** Patient and tumor characteristics of this study

Variables	Overall cohort (*n* = 48)
Age (y)	63.04 ± 9.15
Sex (male/female)	34(73.4%)/14 (26.6%)
Maximum diameter under ultrasonic gastroscopy (mm)	12.9 ± 3.8
Length of muscularis propria in the largest cross‐section of the tumor (mm)	1.22 ± 0.45
Length of muscularis propria + mucosa in the largest cross‐section of the tumor (mm)	2.21 ± 0.71
Maximum diameter of the tumor under CT (mm)	14.58 ± 6.3
Location (u/m/l)	6 (12.5%)/31 (64.6%)/11 (22.9%)
Ultrasound T (2/3)	21 (43.7%)/27 (56.2%)
MRI T (/2/3)	19 (39.5%)/29 (60.4%)
Predict T (/2/3)	15 (31.2%)/33 (68.7%)
Pathology T (/2/3)	22 (45.8%)/26 (54.2%)
Hypertension (yes/no)	13 (20.3%)/35 (79.7%)
Diabetes (yes/no)	2 (4.2%)/46 (95.8%)
Smoking (yes/no)	50 (63.3%)/29 (36.7%)
Drinking (yes/no)	43 (54.4%)/36 (45.6%)
Surgery (open/VATS)	14 (17.7%)/65 (82.3%)
Total length (mm)	6.37 ± 1.92

Abbreviations: CT, computed tomography; MRI, magnetic resonance imaging; VATS, video‐assisted thoracoscopic surgery.

**TABLE 2:** Correspondence of the T stage of each patient under different staging modes

		Pathology T, *n* (%)		Predict T *n* (%)		MRI T *n* (%)		Ultrasound T *n* (%)	
T stage and different modes		T2 22 (45.8)	T3 26 (54.2)	T2 15 (31.2)	T3 33 (68.8)	T2 19 (39.6)	T3 29 (60.4)	T2 21 (43.8)	T3 27 (56.2)
Pathology T	T2 22 (45.8)	–	–	11 (22.9)	11 (22.9)	11 (22.9)	11 (22.9)	11 (22.9)	11 (22.9)
*n* (%)	T3 26 (54.2)	–	–	4 (8.3)	22 (45.8)	8 (16.7)	18 (37.5)	10 (20.8)	16 (33.3)
Predict T	T2 15 (31.2)	11 (22.9)	44 (8.3)	–	–	7 (14.6)	8 (16.7)	7 (14.6)	8 (16.7)
*n* (%)	T3 33 (68.8)	11 (22.9)	22 (45.8)	–	–	12 (25)	21 (43.8)	14 (29.2)	19 (39.6)
MRI T	T2 19 (39.6)	11 (22.9)	8 (16.7)	7 (14.6)	12 (25)	–	–	9 (18.5)	10 (20.8)
*n* (%)	T3 29 (60.4)	11 (22.9)	18 (37.5)	8 (16.7)	21 (43.8)	–	–	12 (25)	17 (35.4)
Ultrasound T	T2 21 (43.8)	11 (22.9)	10 (20.8)	7 (14.6)	14 (29.2)	9 (18.5)	12 (25)	–	–
*n* (%)	T3 27 (56.2)	11 (22.9)	16 (33.3)	8 (16.7)	19 (39.6)	10(20.8)	17 (35.4)	–	–

Abbreviations: MRI, magnetic resonance imaging.

**FIGURE 4:**
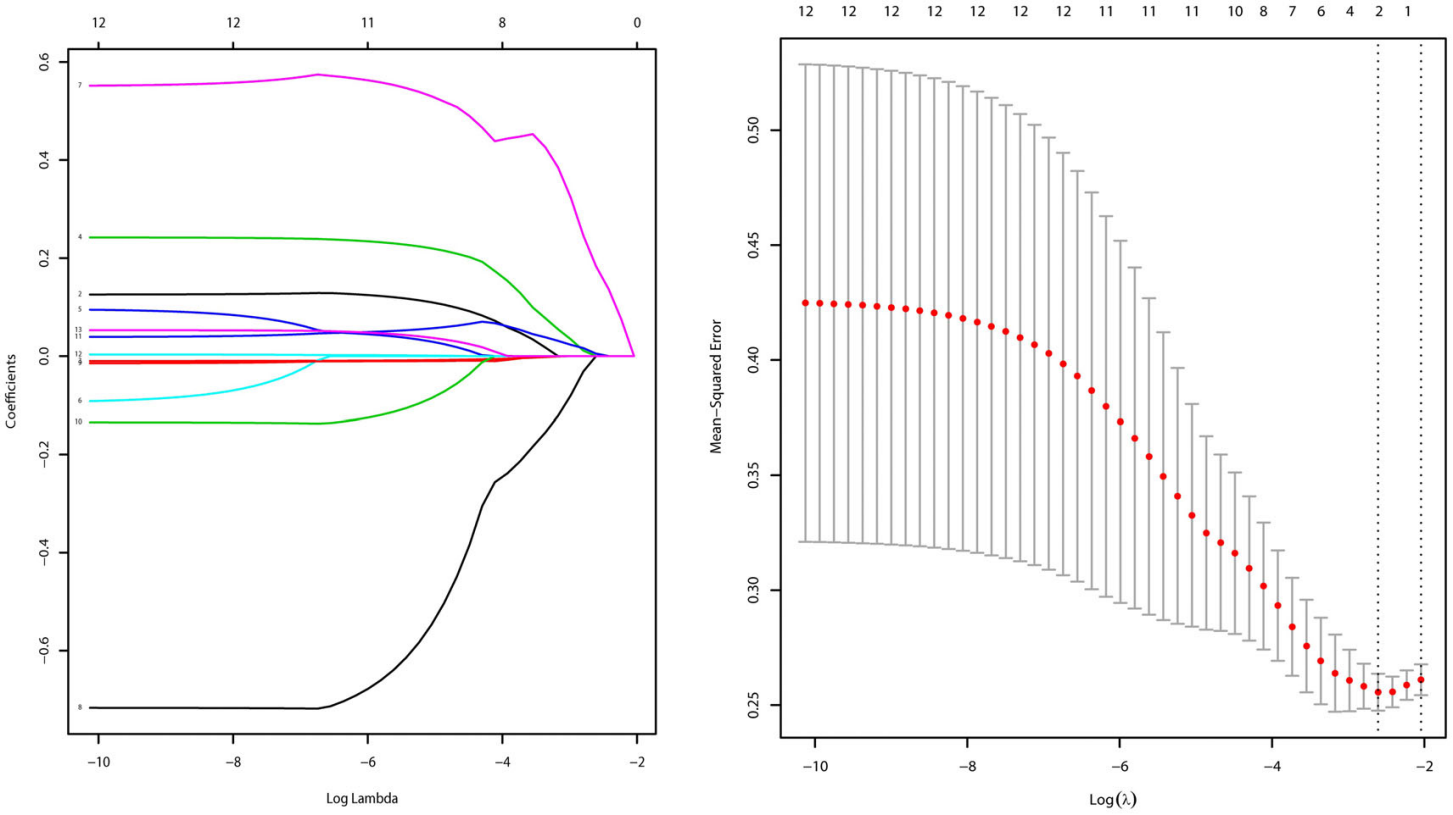
For the screening of predictors, we adopted the least absolute shrinkage and selection operator (LASSO) regression method. (a) LASSO regression was used to pare down the predictors. (b) The penalty coefficients in the LASSO model were adjusted with cross‐validation and the least distance criterion. The vertical black line indicates the best lambda (i.e., the model provided the best fit to the data). The minimal lambda was 0.07369851, with log(λ) = −2.607773.

**FIGURE 5:**
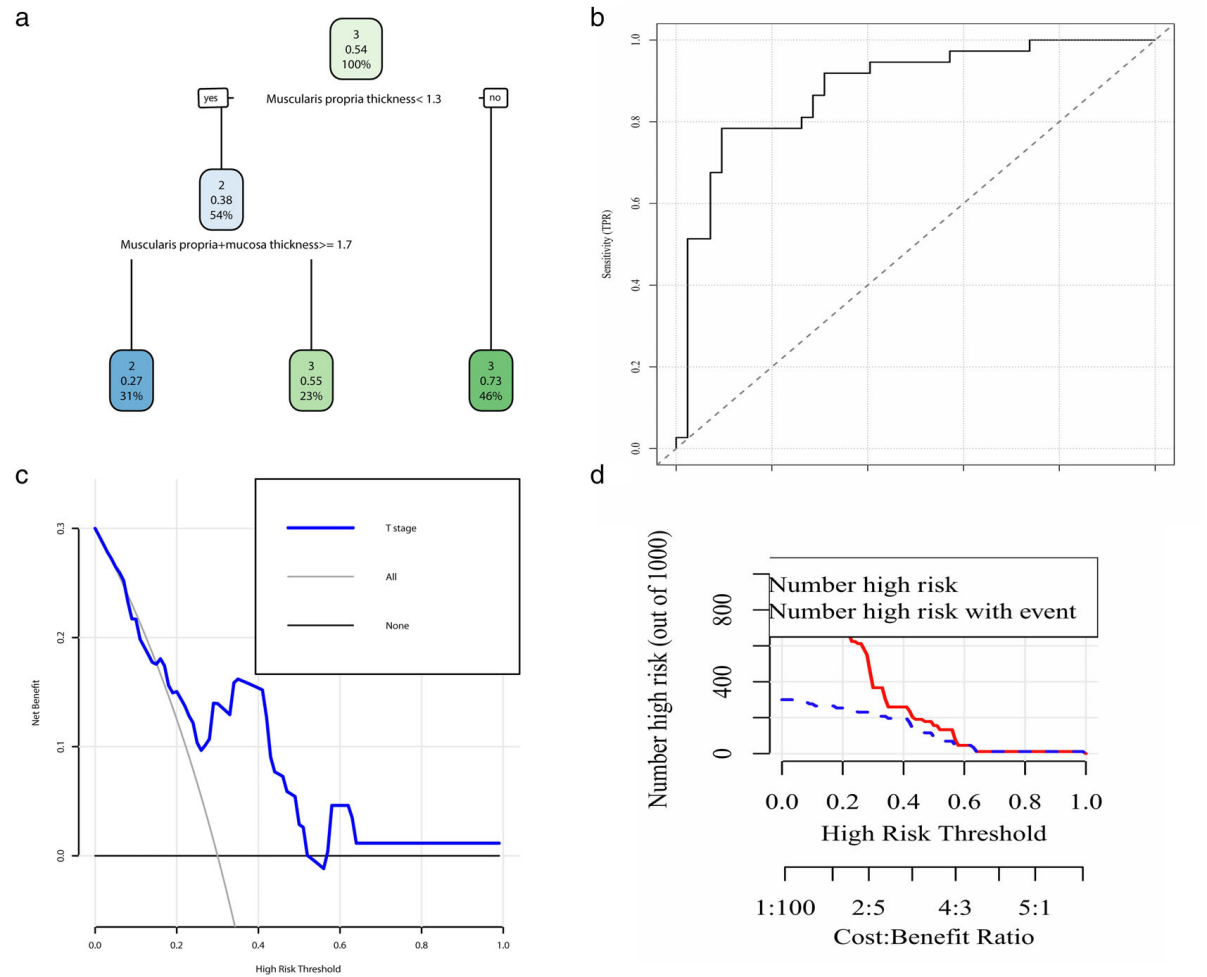
(a) A decision tree was drawn by using the thickness of the muscularis propria in the largest cross‐section of the tumor and the thickness of the muscularis propria + mucosa in the largest cross‐section of the tumor, and the cutoff value was used for determining whether the tumor was T2 or T3. (b) Receiver operating characteristic curve of the model. (c) Decision curve of the model. (d) Clinical impact curve of the model.

As found through LASSO regression analysis, the coefficient λ decreased as the number of variables increased. When λ was optimal, the coefficients of the excluded variables shrank to 0, whereas the variables in the selected model had nonzero coefficients. The results showed that the optimal value of λ was 0.07369851, with log(λ) = −2.607773 (Figure 4). By LASSO reduction, the five clinically relevant factors were reduced to two potential predictors. After multivariate logistic regression analysis, we found that the thickness of the muscularis propria (*p* = 0.0097) and the thickness of the muscularis propria + mucosa in the largest cross‐section of the tumor (*p* = 0.0239) were independent influencing factors. We plotted a decision tree diagram with these two factors (Figure 5(a)). For patients with preoperative cT2 or cT3 tumors, the likelihood of postoperative T3 was 54%. When the thickness of the muscularis propria in the largest cross‐section of the tumor was >1.3 mm, it could be judged as pT3, but when the thickness of the muscularis propria in the largest cross‐section of the tumor was ≤1.3 mm, the thickness of the muscularis propria + mucosa in the largest cross‐section of the tumor should be considered. When the latter was ≥1.7 mm, it could be judged as pT2, otherwise pT3 (Figure 5(a)).

The receiver operating characteristic curve of the model is shown in Figure 5(b). We validated the accuracy of the prediction model, with a Brier score of 0.20 and a C‐index of 0.77 (95% confidence interval [CI], 0.80–0.96). We also plotted the decision curve (Figure 5(c)) and the clinical impact curve (Figure 5(d)) to evaluate the prediction model. Of the tumors diagnosed as T2 by MRI, eight of them were deemed T3 after surgery, and of those deemed T3 by MRI, 11 were deemed T2 after surgery. MRI was accurate in 29/48 cases (60.4%). By ultrasonography, 10 predicted to be T2 were deemed T3 after surgery, and 11 predicted as T3 were deemed T2 after surgery. Ultrasonography was accurate in 27/48 cases (56.3%). By the prediction model, 11 tumors were T2 before and after surgery, and 22 were T3 before and after surgery, for an accuracy of 68.8% (33/48). The model predicted 11 tumors to be T3 that were T2 after surgery, of which eight had no lymph node metastasis and three had lymph node metastasis. It only predicted four tumors to be T2 that were shown to be T3.

**FIGURE 6:**
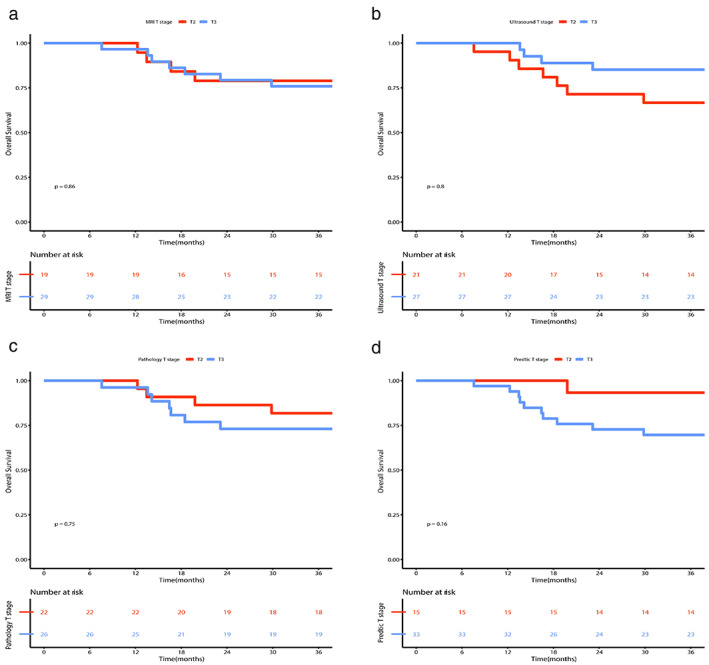
Survival analysis with T stages obtained with grouping by different examination modes. (a) There was no significant difference (*p* = 0.86) between T2 and T3 patient survival when diagnosed by magnetic resonance imaging (MRI), and the survival curves basically coincided. (b) There was no significant difference (*p* = 0.8) between T2 and T3 patient survival when diagnosed by ultrasound, although the T3 group tended to have a better prognosis than the T2 group. (c) There was no significant difference (*p* = 0.75) between T2 and T3 patient survival when diagnosed by postoperative pathology, although the T2 patients tended to fare better than the T3 group. (d) After grouping according to the predicted T stage of our model, the prognosis of patients in T2 stage tended to be significantly better than that of T3 patients, and the *p* value decreased to 0.16.

According to the T stages predicted under different examinations, we performed corresponding survival analysis (Figure 5). These methods showed no significant difference in the grouping analysis of the four T stages. However, in the pathological T stage group, the survival trend of patients with T2 was better than that of patients with T3. Survival was found to be worse in patients with T2 than in patients with T3 after grouping analysis according to ultrasound T stage. The survival curves of stage T2 and T3 patients basically coincided with each other when grouped by MRI staging. After training our prediction model on the determined T stages, the predicted T stages were obtained, and then stratified analysis was performed. Although the results were not significantly different between groups, the T2 and T3 curves were farther from each other than they were with the previous three grouping methods (Figure 6).

## DISCUSSION

From the results, we find that the thickness of the muscularis propria in the largest cross‐section of the tumor and the thickness of the muscularis propria + mucosa in the largest cross‐section of the tumor were independent influencing factors of pathological T stage. Based on these two values, the pathological T stage after surgery could be predicted with 68.8% accuracy.

For patients with locally advanced esophageal cancer, neoadjuvant therapy can improve their prognosis.^1^, ^2^, ^3^, ^4^, ^5^, ^6^, ^7^ According to the latest version of the NCCN guidelines, for patients with clinical cT2N0M0, if the tumor is <3 cm and well differentiated, direct surgical treatment is recommended. If the tumor is >3 cm and poorly differentiated, neoadjuvant chemoradiotherapy followed by surgical treatment or radical chemoradiotherapy is advised. For patients with cT3N0M0, direct surgical treatment is not recommended, but they should be treated under the same principle as patients with >3‐cm poorly differentiated cT2N0M0 (i.e., neoadjuvant therapy followed by surgical treatment or radical chemoradiotherapy).

Our results showed that 39.6% of MRI T stages were inconsistent with the postoperative T stages, and this rate was 43.7% for ultrasound. The treatment regimen may vary because of inaccurate staging, especially for patients with T3N0, in whom the treatment strategy is completely different. Therefore, the evaluation or prediction of T stage is particularly important, because the treatment principles of cT2N0M0 and cT3N0M0 differ greatly and will also differently affect the prognosis. Our prediction model, on the other hand, can effectively predict T stage (with a prediction accuracy of 68.8%), providing a reference for developing better treatment strategies for these patients.

There are a variety of methods for T staging. Common methods are MRI, ultrasonic gastroscopy, positron‐emission tomography–CT, endoscopic mucosal resection, and endoscopic submucosal dissection. Each has its strengths and weaknesses and may be a supplement to other staging methods. There are also new staging methods being reported. Zhou et al.^12^ found that low‐dose spectral insufflation CT protocol combined with automatic spectral imaging assist (GSI assist) can effectively realize the differential diagnosis of stage T1/2 versus stage T3 cancer, with a sensitivity of 56.25% and a specificity of 73.68%. Patients in stage T1 can be well distinguished from those in stage T2 and T3 by ultrasound gastroscopy, but the patients in stages T2 and T3, especially those with slightly larger T2‐stage tumors, may not necessarily be well distinguished. Large intraluminal tumors do not necessarily have a higher T stage than small medullary tumors. The reported diagnostic accuracy of MRI for T1, T2, T3, and T4 esophageal cancer is 33%, 58%, 96%, and 100%, respectively, and the accuracy of T3 staging was significantly higher than that of our study, but the accuracy of T2 staging was not as high as that of T3.^13^ The accuracy of ultrasound is ~64%.^14^, ^15^

The muscularis propria and mucosa are important parts of the esophageal wall, with blood vessels, lymphatic vessels, and nerves running inside it. What will happen to the structures around the tumor after the tumor grows? In particular, what will happen to the lymphatic vessels, because lymphatic metastasis is the main metastasis route of esophageal squamous‐cell carcinoma? Some studies have found that patients with esophageal cancer have significantly more lymphatic plexi in and adjacent to the tumor than patients with no tumor.^8^, ^9^, ^10^ The number of lymphatic plexi in the mucosa adjacent to the tumor is an independent predictor of overall survival in patients with esophageal cancer (hazard ratio, 2.06; *p* = 0.0049).^11^ We infer that with the change in lymphatic plexus number, the thickness of the muscularis propria and that of muscularis propria + mucosa may change to a certain degree, which may also reflect the degree of tumor progression to some extent. When we studied these two thicknesses, we found that both were independent influencing factors of the postoperative differentiation of T2 and T3 tumors. Moreover, a preliminary interpretation of T stage according to our trained values was performed to predict T stage, and the accuracy of this T staging was 68.8% (33/48), which was higher than that of any imaging examination alone.

Our results suggest that after grouping by the predicted T stage and survival analysis, the difference in survival curves of the two groups was greater than that of the grouping by pathological T stage. The *p* value was lower, although still not statistically significant (0.76 vs. 0.16). Eight patients (16.7%) were predicted to have T3 cancer, but eventually were diagnosed with T2N0M0 after surgery, and if these patients were to receive neoadjuvant therapy followed by surgery according to the prediction, then that treatment regimen would not be optimal for them. Similarly, four patients were predicted to have T2 cancer, but were diagnosed with T3 after surgery, so their treatment regimen would also not be optimal.

Our study has limitations, (1) the sample was small, because there were only 48 patients, and it should be expanded for future study. (2) Only the thickness of the muscularis propria and that of the muscularis propria + mucosa were measured in the tumors, and both thicknesses in the normal esophagus (more than 5 cm from the tumor) were not measured, so it could not be determined whether there was a difference in either thickness between the normal esophagus and that with a tumor. (3) Because of the small number of cases, we only performed survival analysis by T stage, without referring to the node and metastasis stages after surgery. Another reason is that the first subjects of our study were patients with cT2‐3N0M0, and the postoperative staging may be inconsistent with the preoperative clinical staging in them. (4) This study only studied the patients that gastroscopy could pass through the esophageal lumen, so it was not applicable for the patients that gastroscopy could not pass through the esophageal lumen.

## CONCLUSIONS

The thickness of the muscularis propria in the largest cross‐section of the tumor and the thickness of the muscularis propria + mucosa in the largest cross‐section of the tumor are independent predicting factors of pathological T stage.

## CONFLICT OF INTEREST

The authors have no conflicts of interest to declare.
